# Remodeling of Liver and Plasma Lipidomes in Mice Lacking Cyclophilin D

**DOI:** 10.3390/ijms231911274

**Published:** 2022-09-24

**Authors:** Balazs Koszegi, Gabor Balogh, Zoltan Berente, Anett Vranesics, Edit Pollak, Laszlo Molnar, Aniko Takatsy, Viktoria Poor, Matyas Wahr, Csenge Antus, Krisztian Eros, Laszlo Vigh, Ferenc Gallyas, Maria Peter, Balazs Veres

**Affiliations:** 1 Department of Biochemistry and Medical Chemistry, Medical School, University of Pecs, 7624 Pecs, Hungary; 2 Institute of Biochemistry, Biological Research Centre, Eötvös Loránd Research Network, 6726 Szeged, Hungary; 3 Research Group for Experimental Diagnostic Imaging, University of Pecs Medical School, 7624 Pecs, Hungary; 4 Department of Comparative Anatomy and Developmental Biology, Institute of Biology, Faculty of Natural Sciences, University of Pecs, 7624 Pecs, Hungary; 5 Ecophysiological and Environmental Toxicological Research Group, Balaton Limnological Research Institute, Eötvös Loránd Research Network, 8237 Tihany, Hungary; 6 Institute of Bioanalysis, Medical School, University of Pecs, 7624 Pecs, Hungary; 7 Szentágothai Research Centre, University of Pecs, 7624 Pecs, Hungary; 8 ELKH-UP Nuclear-Mitochondrial Interactions Research Group, 1245 Budapest, Hungary

**Keywords:** cyclophilin D, lipid metabolism, mitochondrial permeability transition pore, lipidomics, mitochondria, liver, plasma

## Abstract

In recent years, several studies aimed to investigate the metabolic effects of non-functioning or absent cyclophilin D (CypD), a crucial regulatory component of mitochondrial permeability transition pores. It has been reported that the lack of CypD affects glucose and lipid metabolism. However, the findings are controversial regarding the metabolic pathways involved, and most reports describe the effect of a high-fat diet on metabolism. We performed a lipidomic analysis of plasma and liver samples of CypD-/- and wild-type (WT) mice to reveal the lipid-specific alterations resulting from the absence of CypD. In the CypD-/- mice compared to the WT animals, we found a significant change in 52% and 47% of the measured 225 and 201 lipid species in liver and plasma samples, respectively. The higher total lipid content detected in these tissues was not accompanied by abdominal fat accumulation assessed by nuclear magnetic resonance imaging. We also documented characteristic changes in the lipid composition of the liver and plasma as a result of CypD ablation with the relative increase in polyunsaturated membrane lipid species. In addition, we did not observe remarkable differences in the lipid distribution of hepatocytes using histochemistry, but we found characteristic changes in the hepatocyte ultrastructure in CypD-/- animals using electron microscopy. Our results highlight the possible long-term effects of CypD inhibition as a novel therapeutic consideration for various diseases.

## 1. Introduction

Cyclophilin D (CypD) is a mitochondrial peptidyl prolyl-cis,trans-isomerase [1]. It plays a crucial role in mitochondrial permeability transition pore (mPTP) assembly. mPTP opening causes the collapse of the mitochondrial membrane potential, leading to cell death and reactive oxygen species formation [2]. The importance of the increased or decreased incidence of mPTP opening has been implicated in various pathological conditions, such as ischemia-reperfusion injury, neurodegeneration and cancer development [3,4,5]. mPTPs are considered to have a Ca^2+^ channel function; their transient opening may modulate mitochondrial energetics [6,7,8]. In addition, CypD has been recently identified as a candidate for a mitochondrial partner of the mitochondria-associated membrane (MAM), in which the endoplasmic reticulum (ER) and mitochondria are in physical contact [9,10]. These organellar contact sites play a role in the Ca^2+^ and phospholipid exchange between the ER and the mitochondrion and could be affected in various diseases.

Based on the evidence that CypD has the abovementioned roles, several studies aimed to reveal the physiological and metabolic changes in the absence of the functioning enzyme. The invalidation of CypD by pharmacological or genetic methods prevents mPTP opening and protects against myocardial and brain reperfusion injury [11,12,13,14,15]. Moreover, the lack of CypD mitigates the lipopolysaccharide-induced inflammatory response, as was described by our group previously [16,17]. Other studies focused on the effect of CypD disruption on metabolic homeostasis. The impact of CypD deficiency on lipid metabolism and obesity has been investigated widely in recent years with controversial results. Previous studies reported on CypD knock-out (CypD-/-) mice with adult-onset obesity and lipid accumulation on a normal and a high-fat diet [18,19]. On the contrary, other studies found unaltered body weight [20] or prevented hepatic steatosis on a high-fat diet [21]. Furthermore, it has been observed that the loss of functioning CypD resulted in changes in branched-chain amino acid degradation pathway, citric acid cycle and pyruvate metabolism in the heart [22], and decreased fatty acid oxidation with enhanced glycolytic activity in the liver [23].

In addition, the implication of mPTP opening in various diseases has drawn attention to the pore components as therapeutic targets [24,25]. Although the constituents of mPTPs are still under scientific debate, CypD is a widely accepted regulator of pore opening [26]. Thus, it is highly desirable to elucidate the metabolic changes due to CypD ablation. Since the dysregulation of the lipid metabolism is concerned in several diseases and various lipid species have a vital role in cell signaling, we performed a lipidomic analysis of plasma and liver samples of CypD-/- mice to describe the alterations regarding the lipid species content and composition in these animals. We also used magnetic resonance imaging (MRI), histochemistry and conventional transmission electron microscopy (TEM) to investigate the lipid accumulation in CypD-/- mice and reveal the histological and ultrastructural characteristics of hepatocytes.

## 2. Results

### 2.1. Significant Remodeling of the Liver and Plasma Lipidomes Occurred in Response to CypD Deletion

**Figure 1:**
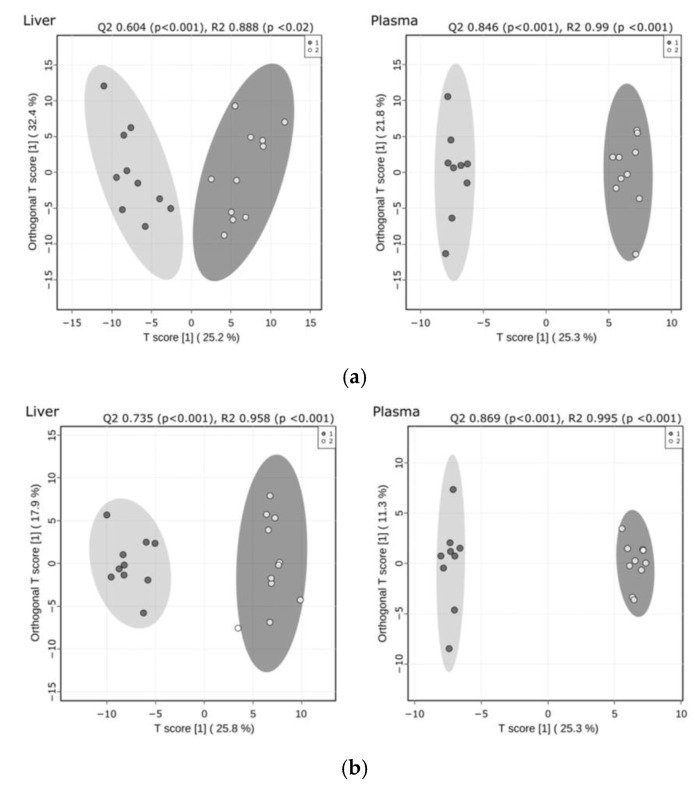
OPLS-DA shows the separation of WT (black circles) and CypD-/- (open circles) lipidomes. The analysis was performed using (**a**) absolute (lipid nmol/liver mg or lipid nmol/plasma mL) and (**b**) relative (mol% of polar lipids) lipid concentrations. Permutation tests (*n* = 1000) revealed a good predictability (Q2) and high goodness of fit (R2) as indicated.

To identify lipid biomarker signatures due to CypD ablation, we carried out high sensitivity, high-resolution shotgun mass spectrometry (MS)-based lipidomic measurements. We identified and quantitated 225 and 202 lipid species encompassing 23 and 18 lipid classes in liver and plasma samples, respectively, including membrane/polar, signaling and storage lipids. To present the lipidomic data, we calculated absolute quantities (lipid nmol/liver mg or lipid nmol/plasma mL) and relative levels (mol% of polar lipids) (Tables S1–S4). Absolute and relative lipid concentration values were subjected to Orthogonal Projections to Latent Structures Discriminant Analysis (OPLS-DA) (Figure 1). The WT and CypD-/- samples were separated into well distinguished clusters, indicating that characteristic changes occurred both in the content and composition of the liver and plasma lipidomes as a result of CypD ablation. Group separations validated by permutation tests revealed a good predictability (Q2) and high goodness of fit (R2).

### 2.2. The Lipid Content Increased Due to CypD Ablation Both in the Liver and Plasma

**Figure 2:**
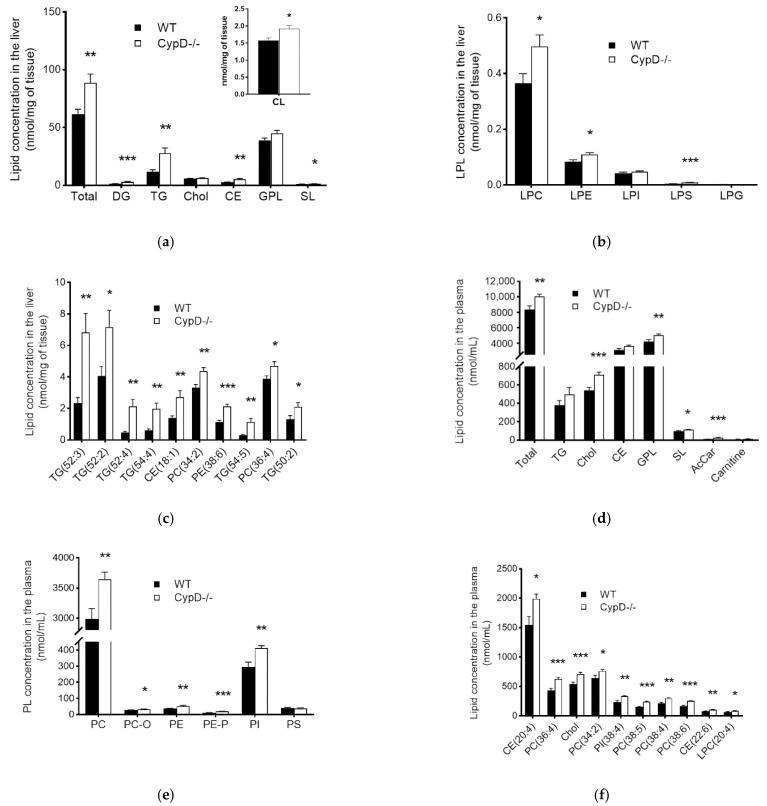
Comparison of the absolute lipid content of WT and CypD-/- samples. Bar charts represent the (**a**) total and the lipid class-based lipid content of liver samples with the measured total cardiolipin in insert, (**b**) LPL lipid content, and (**c**) the concentration of the top ten species that contributed most to the overall lipid accumulation in liver samples, as well as the (**d**) total and lipid class-based lipid content of plasma samples, (**e**) PL lipid content and (**f**) the concentration of the top ten species that contributed most to the overall lipid accumulation in plasma samples. Data are shown as mean ±SEM, *n* = 9–11, * *p* < 0.05, ** *p* < 0.01, *** *p* < 0.001. AcCar: acylcarnitine, Chol: cholesterol, CE: cholesteryl ester, CL: cardiolipin, DG: diacylglycerol, GPL: glycerophospholipid, LPC: lisophosphatidylcholesterol, LPE: lisophosphatidylethanolamine, LPG: lisophosphatidylglycerol, LPI: lisophosphatidylinositol, LPL: lysophospholipid, LPS: lisophosphatidylserine, PC: diacyl phosphatidylcholine, PC-O: alkyl-acyl phosphatidylcholine, PE: diacyl phosphatidylethanolamine, PE-P: alkenyl-acyl phosphatidylethanolamine, PI: phosphatidylinositol, PL: phospholipid, PS: phosphatidylserine, SL: sphingolipid, TG: triacylglycerol.

We detected a significant 44% increase in the total lipid content of the liver in CypD-/- mice compared to the WT (Figure 2a); altogether, 117 out of 225 quantitated species (52%) were elevated (Table S1). At the lipid class level, remarkable changes occurred in storage lipids, triacylglycerols (TG) and cholesteryl esters (CE), as well as in diacylglycerols (DG) (Figure 2a and Table S1). Although the sum level of glycerophospholipids (GPLs) did not change, we observed a significant accumulation in cardiolipin (CL) (Figure 2a, insert), the hallmark lipid of mitochondria. The concentrations of lysophosphatidylcholine (LPC), lysophosphatidylethanolamine (LPE) and lysophosphatidylserine (LPS) were also elevated (Figure 2b and Table S1). The sum amount of sphingolipids increased slightly due to an increasing tendency in sphingomyelin (SM, *p* = 0.051), the major sphingolipid class, as well as in lactosylceramide (LacCer, *p* = 0.021), whereas the absolute concentration of cholesterol (Chol) did not change (Figure 2b and Table S1). At the lipid species level, we identified six TG species among the top 10 components that contributed the most to the overall lipid accumulation, along with CE, phosphatidylcholine (PC) and phosphatidylethanolamine (PE) species (Figure 2c).

In plasma samples, we also observed a remarkable 20% accumulation in the total lipid amount (Figure 2d), which arose from the elevation of 95 out of 201 analyzed components (47%) (Table S2). This elevation is distributed among GPLs, sphingolipids (SLs), Chol and acylcarnitines (Figure 2d and Table S2). The increased GPL level results from the significant increase in PCs (both diacyl and alkyl-acyl), PEs (both diacyl and alkenyl-acyl) and phosphatidylinositols (PI) (Figure 2e and Table S2). Nevertheless, at the lipid species level, we found PC, CE, TG and LPC species among the top 10 elevated components (Figure 2f).

### 2.3. Lipid Composition Changed Due to CypD Ablation

**Figure 3:**
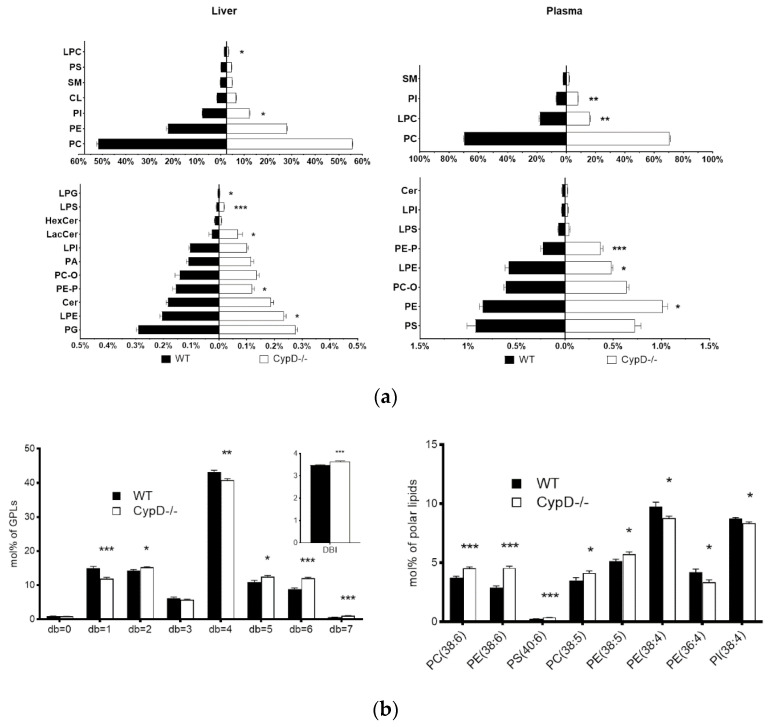
Comparison of the relative lipid content of WT and Cypd-/- samples. Bar charts represent the (**a**) phospholipid composition in liver and plasma samples and (**b**) the double bond number with the calculated DBI in the liver membrane lipidome and the relative concentrations of selected PL species. Data are shown as mean ±SEM, *n* = 9–11, * *p* < 0.05, ** *p* < 0.01, *** *p* < 0.001. Cer: Ceramide, CL: cardiolipin, HexCer: hexosylceramide, LacCer: lactosylceramide, LPC: lisophosphatidylcholesterol, LPE: lisophosphatidylethanolamine, LPG: lisophosphatidylglycerol, LPI: lisophosphatidylinositol, LPS: lisophosphatidylserine, PA: phosphatidic acid, PC: diacyl phosphatidylcholine, PC-O: alkyl-acyl phosphatidylcholine, PE: diacyl phosphatidylethanolamine, PE-P: alkenyl-acyl phosphatidylethanolamine, PG: phosphatidylglycerol, PI: phosphatidylinositol, PS: phosphatidylserine, SM: sphingomyelin.

To rank the effect of CypD ablation on the lipidome composition, we calculated the sum of the absolute mol% difference (SoamD) score [27] relative to the WT. SoamD score accounted for 31% and 15% in the liver and plasma, respectively, corresponding to a sizeable lipidome reshaping in both tissues. Based on the mol% of polar lipid values, in the liver of CypD-/- mice compared to WT animals, 120 out of 225 quantitated species changed significantly (53%, Table S3), whereas this number was 101 out of 201 in the plasma (50%, Table S4) of these animals. The phospholipid composition of samples showed notable alteration. Compared to WT mice, in the liver samples of CypD-/- animals, we detected a relative increase in LPC, LPE, LPS and LacCer, and a relative decrease in PI, PE-P and LPG. We also noted that, although the relative concentration of CL did not change, its species profile shifted toward a significantly higher symmetric tetralinoleoyl species (CL (72:8)) content (Table S3). In the plasma samples of CypD-/- animals, the relative concentration of PE, PE-P and PI increased, whereas those of LPC and LPE decreased (Figure 3a). 

Another remarkable consequence of this remodeling was the increase in the double bond index (DBI) of the liver membrane lipidome. The DBI is a measure of unsaturation, and its elevation derives largely from the relative increase in membrane lipid components with a double bond number (db) = 5 and 6, and a concomitant reduction in monounsaturated species (db = 1) (Figure 3b). Lipid species with db = 5 contain the polyunsaturated omega-6 arachidonic acid (20:4, AA) in combination with a monounsaturated fatty acid (16:1 or 18:1), such as PC (38:5, 18:1/20:4) or PE (38:5, 18:1/20:4), whereas those with db = 6 contain the omega-3 docosahexaenoic acid (22:6, DHA) in combination with a saturated fatty acid (16:0 or 18:0), such as PC (38:6, 16:0/22:6) or PS (40:6, 18:0/22:6). The most abundant lipid pool of the liver membrane is represented by species with db = 4. These lipids contain AA in combination with a saturated fatty acyl (16:0 or 18:0), e.g., PE (38:4, 18:0/20:4) or PI (38:4, 18:0/20:4) (Figure 3b). Interestingly, the relative concentration of this pool decreased, and therefore, the ratio of DHA/AA increased (Table S3). Furthermore, it was paralleled with a significant increase in AA-containing lysophospholipid (LPL) species (Table S3). In the liver sphingolipid pool, the most remarkable change was the remodeling of sphingomyelin (SM), characterized by a significant increase in the major 24:1-fatty acid-containing species SM (42:2:2), whereas the relative concentration of cholesterol did not change (Table S3).

In plasma GPLs, we detected a significant accumulation in the relative concentration of polyunsaturated species (db ≥ 4), but unlike in the liver, species with db = 4 were also elevated (Table S4). The level of acylcarnitines, especially that of acetylcarnitine (AcCar (2:0)), displayed a significant enhancement in the plasma lipidome at the compositional level as well (Table S4).

### 2.4. The Level of Several Liver and Plasma Phospholipid Species Showed a Positive Correlation

**Figure 4:**
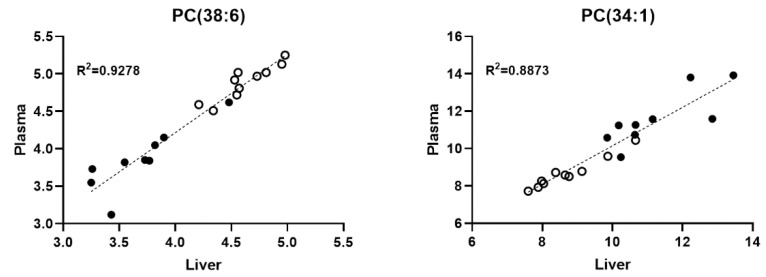
Correlation between the liver and the plasma relative lipid concentrations for PC (38:6) and PC (34:1). Black: WT samples, open: CypD-/- samples. Mol% of polar lipid values were correlated using the Pearson correlation.

Under normal conditions, most of the plasma phospholipids are secreted by the liver [28]. Therefore, any alteration in the liver function that affects lipid metabolism might interfere with plasma lipidomic patterns. Accordingly, we analyzed the potential correlations between the liver and plasma lipidomes using the Pearson correlation. We found strong (r > 0.7) correlations for several species, e.g., for PC (38:6) and PC (34:1) (Figure 4 and Table S5).

### 2.5. CypD Ablation Did Not Result in Abdominal Fat Accumulation

**Figure 5:**
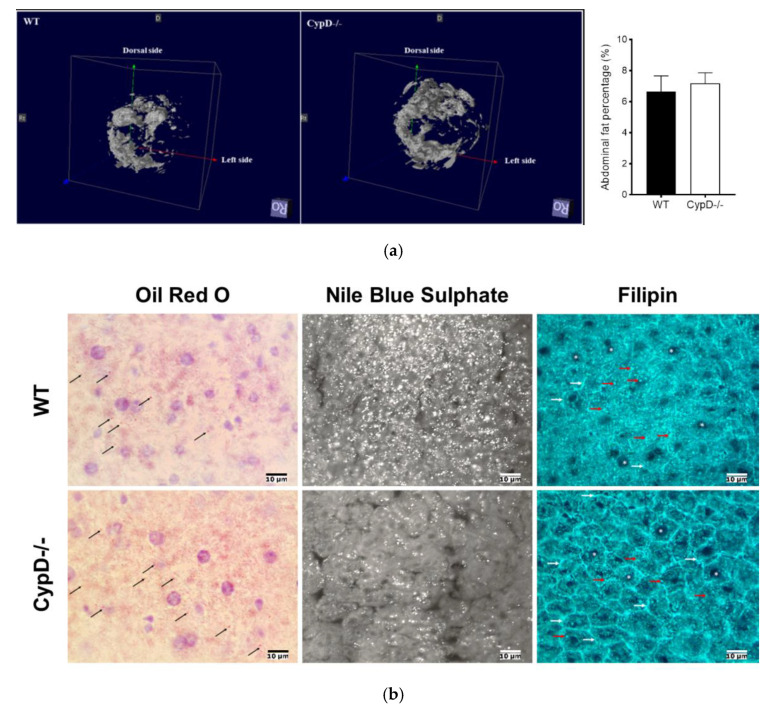
In vivo body fat measurements and histological analysis of liver lipid distribution in WT and CypD-/- mice. The in vivo abdominal fat distribution of WT and CypD-/- mice (*n* = 6) were compared using MRI (T1 3D FLASH sequence with and without magnetization transfer calibrated to a water–fat phantom). (**a**) Representative images show the 3D distribution of abdominal fat (resolution: 120 × 120 × 2250 μm^3^); bar chart shows the statistical analysis of body fat distribution between WT and CypD-/- animals. Data are shown as mean ± SEM, *n* = 10. (**b**) The occurrence and distribution pattern of neutral lipids (Oil Red O), phospholipids and fatty acids (Nile Blue Sulphate), and cholesterol (Filipin) in the liver cells of WT and CypD-/- animals. Arrows show lipid droplets, and asterisks label the cell nuclei.

Due to the elevated levels of storage lipids (in the liver) and cholesterol (in the plasma), we assumed an increased body fat accumulation in CypD-/- mice. We performed MRI analysis to measure the amount of abdominal fat, which did not reveal a significant difference between WT and CypD-/- animals (Figure 5a). The cholesterol content of feces was also measured and showed no significant difference either (Figure S1).

There were no marked differences in the histological organization of midgut, kidney and liver of WT and CypD-/- animals (not shown). Since the liver plays a central role in lipid metabolism and turnover, further investigations focused on its histological, histochemical and ultrastructural characteristics. Histological observations analyzing the size and shape of hepatocytes, organization of their nucleus and cytoplasm and the pattern of its circulation (namely portal triads, sinusoids and central veins) did not find any differences between WT and CypD-/- animals (not shown).

The distribution pattern of neutral lipids (Oil Red O staining), phospholipids and fatty acids (Nile Blue Sulphate staining), and free cholesterol (Filipin staining) showed similar characteristics in both animal groups. Small neutral lipid droplets with a random appearance were seen in the cytoplasm of hepatocytes (Figure 5).

### 2.6. TEM Investigation Revealed Structural Differences between CypD-/- and WT Hepatocytes

**Figure 6:**
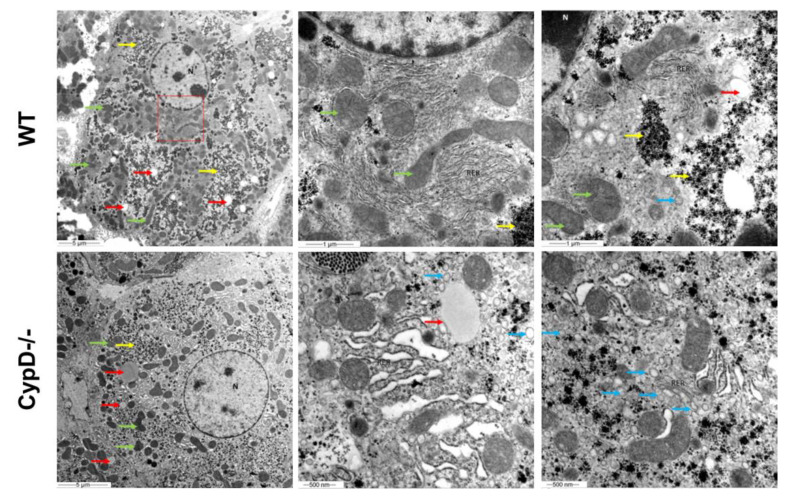
Observations on the fine structures of hepatocytes. N: nuclei, RER: rough endoplasmic reticulum, green arrows: mitochondria, yellow arrows: glycogen granules, red arrows: lipid droplets, blue arrows: vesicles.

Although no difference in the lipid distribution was observable with the histochemical analysis, characteristic alterations were seen between the ultrastructure of WT and CypD-/- hepatocytes, the arrangement of glycogen particles and lipid droplets, the distribution of mitochondria, the extension and organization of rough endoplasmic reticulum (RER) cisternae and activity of the Golgi dictyosomes. The cytoplasm of the control hepatocytes was denser than CypD-/- ones due to the large parts filled up with glycogen particles and the higher number of mitochondria with dense matrix scattered over. According to the histochemical findings, a few lipid droplets with variable sizes were located in the hepatocytes. A characteristic difference was seen in the organization of RER cisternae as well. In the WT samples, hepatocytes were extended and narrow RER cisternae occurred, while in the CypD-/- ones, fragmented, enlarged cisternae were seen. A marked structural difference was the presence of more active Golgi dictyosomes in the latter hepatocytes, resulting in an accumulation of secretory vesicles, filled with finely granulated material, in the whole cytoplasm. The chemical composition of the secretory product has not been identified yet. The mitochondria of the WT mice established large groups formed with intermingled RER cisternae. In contrast, in the CypD-/- animals, these formations were far less characteristic, the order was loosened up, and the dilated RER cisternae was observed in the vicinity of mitochondria. Compared to WT hepatocytes, CypD-/- cells comprised mitochondria with a dense matrix and in a more dispersed arrangement (Figure 6).

## 3. Discussion

In this report, we described the effect of CypD deletion on the lipidomes of plasma and the liver of mice. Mass spectrometry-based quantitative lipidomic analysis revealed extensive alterations in the lipid content and lipid profile in CypD-/- mice compared to WT littermates. Moreover, we showed that this remarkable change is not accompanied by abdominal fat accumulation.

Although CypD has been investigated thoroughly since its purification [1], the supposed relation of this mitochondrial protein to cell metabolism is still obscure. In addition to the implication of CypD in mPTP opening, several proteins have been reported as binding partners of this mitochondrial peptidyl prolyl-cis,trans-isomerase [26]. Furthermore, the role of CypD in mitochondrial function has been studied and reported. The interaction of CypD with mitochondrial transcription factors can affect mitochondrial RNA synthesis, thus altering the expression of subunits in electron transport chain and ATP synthase. The lack of CypD can affect cell proliferation and motility via retrograde signaling and transcriptional changes [29]. The growing number of identified binding partners highlights the possible roles of CypD in cell metabolism. According to this, the effect of CypD deletion on metabolic homeostasis has been studied in recent years. A report based on cardiac mitochondrial proteome analysis showed that lack of CypD resulted in changes in the levels of several proteins. According to pathway analysis, these changes occurred in the branched-chain amino acid degradation pathway, citric acid cycle and pyruvate metabolism [22]. In the same study, decreased carnitine palmitoyltransferase I activity was observed with decreased long-chain acylcarnitine levels, suggesting reduced beta-oxidation, which is consistent with previous findings that suggested a metabolic shift from fatty acid oxidation to glycolysis [30]. Tavecchio et al. found that CypD deletion led to increased levels of acylcarnitines and decreased activity of the mitochondrial trifunctional protein, an enzyme catalyzing the key steps in beta-oxidation [23].

The well distinguished clusters obtained by the multivariate statistical analysis of lipidomic datasets suggested a remarkable reshaping of the liver and plasma lipidomes due to CypD absence. In line with Tavecchio et al., we observed elevated acylcarnitine levels with insignificant carnitine level changes in CypD-/- mice, a possible hallmark of beta-oxidation impairment. The elevation in TG species in the liver might represent a possible fate of fatty acids from the impaired beta-oxidation. Yet, the increase in storage lipids, TG and CE, detected by quantitative lipidomic analysis, was not associated with abdominal fat accumulation, in agreement with a previous report where the tissue-specific ablation of CypD was studied [19]. Because DG and lysolipids are central intermediates in lipid metabolic pathways, their selective accumulation in the liver might indicate an increased lipid synthetic activity in the absence of CypD in the liver. In addition, the elevation in AA-containing LPL species, paralleled with a significant reduction in certain AA-containing fully acylated GPLs, points to an increased phospholipase A1 activity. It was shown that the calcium-induced opening of mPTPs and the subsequent cytochrome c release are diminished by the genetic ablation of calcium-independent phospholipase A2γ [31]. This enzyme exhibits PLA1 activity on phospholipid substrates containing polyunsaturated fatty acids esterified to the sn-2 position, producing saturated fatty acids (from the sn-1 position) and 2-polyunsaturated lysolipid molecular species [32]. Therefore, our observation might represent a compensatory effect in the absence of CypD. 

A further important finding was the elevated level of the characteristic inner mitochondrial membrane glycerophospholipid CL in CypD knock-out animals. CL is a proven stabilizing lipid partner of proteins embedded in the inner mitochondrial membrane, and its structural alteration is involved in various conditions [33]. However, the link between the increased concentration of this phospholipid and the lack of CypD is elusive and requires further research. Furthermore, it has been described that CypD modulates the mitochondria–ER connection through MAM [9,10], and this contact site between mitochondria and ER bears a crucial role in phospholipid biosynthesis and transport and is also implicated in esterification and transport of cholesterol [34]. The relative increase in polyene membrane lipid species in the absence of CypD contributes to a significant increase in membrane fluidity in the liver, as quantitated by the double bond index. Sphingolipids and cholesterol are also known to regulate membrane fluidity. However, the relatively subtle change in the sphingolipid pool with the unaltered cholesterol levels presumably could not compensate for the fluidity increase caused by polyenoic components. Based on these compositional changes in the liver membrane lipidome, we propose that the lack of CypD may disrupt MAM integrity, resulting in perturbed phospholipid composition and affecting ER-localized processes, a possible explanation for the ultrastructural changes we observed in CypD-/- animals. It may also affect the cholesterol metabolism, as shown here with elevated cholesteryl ester levels in the liver and increased cholesterol levels in the plasma due to CypD deletion. Nevertheless, this phenomenon and the possible link between CypD and ER processes through MAM have to be elucidated. On the other hand, the higher amount of polyene membrane lipids and especially the higher DHA/AA ratio might represent an extra pool for generating vital signaling lipids (lysolipids, long-chain free fatty acids, and eicosanoids/docosanoids) under pathophysiological conditions, such as in an inflammatory response.

Our results reveal a comprehensive reshaping in the lipidomic profile of the CypD-/- mice compared to their WT littermates, highlighting the possible long-term metabolic and structural effects of CypD inhibition. These changes in the lipidome may lead to perturbed membrane composition, membrane–membrane interaction and signal transduction and may affect the mitochondrial structure and metabolism. Moreover, we found positive correlations between the levels of various phospholipid species in the liver and plasma. The alteration of the plasma lipidomic pattern presumably affects the lipidome of other tissues, which utilize circulating fatty acids to build up their lipids and membranes. The relative increase in polyunsaturated components might represent a beneficial long-term consequence on the function of such organs as a result of CypD inhibition [35]. 

The opening of mPTPs is involved in several diseases. Ischaemia-reperfusion injury is a well-documented mitochondrial permeability transition-related condition with Ca^2+^ overload and oxidative stress as inducers of the pore opening. Ca^2+^ overload and increased levels of reactive oxygen species are also potential inducers of mPTP opening in the mechanism of one type of mitochondria-dependent neuronal cell death. This mechanism has been reported in Parkinson’s and Alzheimer’s diseases. Moreover, the relation between the opening of mPTPs and mitochondrial disorders has been also suggested. Among the consequences of prolonged mPTP opening, the reversal of ATP synthase has been described with ATP loss and perturbed energy metabolism, an underlying process in mitochondrial disorders. Due to the implication of mPTP opening in various conditions, the regulation of pore opening and the components of the pore are possible therapeutic targets [24,25,36,37]. In light of our findings, these therapeutic approaches should consider the aforementioned long-term effects.

## 4. Materials and Methods

### 4.1. Animals

Male 6-month-old wild-type C57BL/6 and Cyclophilin D knock-out mice (CypD-/-) with C57BL/6 background were supplied by Prof. László Tretter (Semmelweis University, Budapest, Hungary). Mice were kept under standard conditions according to the regulations of the National Institutes of Health (NIH). Food and tap water were provided ad libitum. All animal experiments were approved by the Institutional Animal Use and Care Committee of the University of Pecs (registration no.: BA02/2000-15/2017) and conformed to the guidelines from Directive 2010/63/EU of the European Parliament on the protection of animals used for scientific purposes.

### 4.2. Lipid Analysis

Lipid standards were from Avanti Polar Lipids (Alabaster, AL, USA) and Cambridge Isotope Laboratories (Tewksbury, MA, USA). Solvents for extraction and MS analyses were liquid chromatographic grade from Merck (Darmstadt, Germany) and Optima LC-MS grade from Thermo Fisher Scientific (Waltham, MA, USA). All other chemicals were of the best available grade, purchased from Sigma-Aldrich (St. Louis, MO, USA) or Thermo Fisher Scientific.

A piece of liver tissue (typically of 100–300 mg) was homogenized in water using a bullet blender homogenizer (Bullet Blender Gold, Next Advance, Inc., Averill Park, NY, USA) in the presence of zirconium oxide beads (0.5 and 1 mm), at a speed level of 10 for 3 min at 4 °C. A portion of the homogenate (corresponding to 2 mg wet weight) or 20 µL plasma was subjected immediately to simple one-phase methanolic lipid extraction [38]. First, the liver homogenate or plasma aliquot was sonicated in 1 mL methanol containing 8 µg di20:0 PC, 6 µg d7-Chol (as extraction standards) and 0.001% butylated hydroxytoluene (as an antioxidant) in a bath sonicator for 5 min, then shaken for 5 min and centrifuged at 10,000 g for 5 min. The supernatant was transferred into a new Eppendorf tube and stored at −20 °C until MS analysis. For MS measurements, 5 µL of liver lipid extract or 15 µL of plasma lipid extract was diluted to 150 µL with an infusion solvent mixture (chloroform:methanol:iso-propanol 1:2:1, by vol.) containing an internal standard mix (Table S6). Next, the mixture was halved, and 5% dimethylformamide (additive for the negative ion mode) or 4 mM ammonium chloride (additive for the positive ion mode) was added to the split sample halves. Mass spectrometry analyses were performed on an Orbitrap Elite instrument (Thermo Fisher Scientific, Bremen, Germany) equipped with a TriVersa NanoMate robotic nanoflow ion source (Advion BioSciences, Ithaca, NY, USA) as described previously [38]. Lipid species were identified by LipidXplorer 1.2.8.1 software [39]. Identification was executed by matching the *m/z* values of their monoisotopic peaks to the corresponding elemental composition constraints. Mass tolerance was set to 2 ppm. To resolve fatty acyl composition in glycerol (phospho) lipids, data-dependent tandem MS2 or MS3 fragmentation experiments were performed based on mass lists from survey scans as in [40]. Data files generated by LipidXplorer queries were processed further by in-house Excel macros. To annotate lipid classes and species, we applied the classification systems for lipids [41]. Sum formulas for glycero(phospho)lipids are defined as the lipid class abbreviation followed by the total number of carbons and the total number of double bonds for all chains, e.g., PC (36:4) or TG (54:3).

Lipidomic data were expressed as both absolute concentrations (lipid nmol/liver mg or lipid nmol/plasma mL) and relative concentrations (mol% of polar lipids, where polar lipids include all quantitated lipids except DG, TG, CE, Chol, carnitine and acylcarnitines).

SoamD score, the sum of absolute mol% differences, was calculated for liver and plasma samples as SoamD = Σ (abs ([Spec *_(i,KO)_*] − [Spec *_(i,WT)_*])), where [Spec *_(i,KO)_*] indicates the mol% of lipid species *i* in the CypD knock-out mice, [Spec *_(i,WT)_*] indicates the mol% of lipid species *i* in the WT mice, and mol% is expressed as a percentage of the sum of all quantitated lipid species.

The double bond index was calculated for fully acylated glycerophospholipids (GPL) as Σ (db × [GPL *_(i)_*])/Σ [GPL *_(i)_*], where db is the total number of double bonds in fatty acyls in a given GPL species *i*, and the square bracket indicates mol% of GPLs.

### 4.3. MRI Analysis

All MRI acquisitions were performed using a 4.7T small-animal MRI system (Pharmascan 47/16 US; Bruker BioSpin MRI GmbH, Ettlingen, Germany) with a volume RF coil with an inner diameter of 35 mm.

During the MRI measurements, the animals were held under inhalation anesthesia using 1–2% (3% for induction) isoflurane in a 1:2 mixture of O_2_/N_2_O. Respiration was monitored using a respiratory monitoring system and was stable within 30–40 breaths/min range.

After a localizer scan in three directions, axial T1-weighted three-dimensional gradient-echo imaging (T1_FLASH_3D) was performed with the following parameters: TR = 20 ms; TE = 3.105 ms; field of view (FOV) = 32 × 30 mm; matrix = 192 × 192; slice thickness = 36 mm with FOV saturation and triggered to the breathing of the animal. This measurement was repeated with magnetization transfer (MT) in order to suppress the signal originating from water in abdominal tissues using the following parameters: TR = 50 ms; TE = 8.6 ms; field of view (FOV) = 32 × 30 mm; matrix = 256 × 256; slice thickness = 36 mm, MT: irradiation offset: 700 Hz; N.o pulses: 1; interpulse delay: 0.01 ms. Thus, the contribution of water to the MR signal was negligible compared to the contribution of fat. The MT parameters were determined using a mouse-sized phantom made from two concentric tubes, the inner one containing water and the outer one containing pork lard. The imaging experiment covered the whole cross-section of the mice from the diaphragm to the sacrum.

The wild-type male C57BL6 mice fed with standard nutrition (*n* = 6) and cyclophilin D knock-out male C57BL6 mice fed by standard nutrition (*n* = 6) were examined.

Paravision 6.0.1 software was used to manually determine the total volume of the abdominal cavity from the basic T1 FLASH experiment, and then the total volume of adipose tissue was estimated from the images obtained in the MT experiment in all animals. The apparent fat percentage was calculated from these values.

### 4.4. Tissue Preparation for Histological and Histochemical Analysis

The control and CypD-/- mice were anaesthetized, tissue samples were removed, and 1×1 cm pieces of all liver lobes were isolated. Thereafter samples were treated consistently in the same way.

### 4.5. Histochemistry

For the light microscopic observation of the fat component of hepatic tissue, cryosectioned samples were used. To improve the preservation of phospholipids, modified Baker’s formol-calcium solution was used containing 8% formalin supplemented with 10% calcium chloride. Samples then were pre-treated in the oversaturated sucrose solution overnight. This manner of cryopreservation is widely applied to reduce the structural and functional changes resulting from short-term freezing. The 10 µm thick serial sections were cut from fresh frozen blocks, then collected on adhesive medium coated Superfrost (Sigma-Aldrich) glass slides and kept at −20 °C until histological staining. To prove the presence, distribution, ratio and compound nature of lipids, three parallel lipid histochemical methods were performed on frozen liver sections.

Filipin staining was carried out on frozen sections to visualize free cholesterol. First, a stock solution of 2.5 mg Filipin diluted in 1 mL dimethylformamide was prepared. For staining, frozen sections were brought to room temperature equilibrium 0.2 mL Filipin stock solution diluted in 10 mL phosphate-buffered saline (PBS; 0.1 M pH 7.4) was made freshly and overlaid on slides incubated in wet chambers for 30 min in the dark. This was followed with two short rinses in PBS to remove excess stain solution, and then slides were mounted in glycerin jelly buffered with PBS.

To examine the phospholipid content, Nile blue A (Nile blue sulphate; Sigma Aldrich) was selected as Nile blue sulphate is an easy-to-use water-soluble basic oxazine dye. A simple 1% aqueous stock solution of Nile blue was prepared and employed directly to our sections derived from the same cryoprotected frozen tissue and washed in PBS three times for 2 min before. Slides were incubated at room temperature for 20 min under the abovementioned circumstances, and then they were washed in distilled water and mounted in buffered glycerol.

Oil red stain was applied to demarcate neutral triglyceride occurrence and other neutral fat molecules of hepatic tissue. Slides were washed briefly in distilled water and then rinsed in 70% isopropyl alcohol. Saturated Oil red stock solution in 70% isopropyl alcohol was applied to the slides directly, and samples were incubated for 30 min as written above. Following a quick rinse in 70% alcohol for differential staining, three changes of distilled water and a 6-minute-long alum hematoxylin stain were carried out, resulting in good nuclear appearance to help with cellular level analysis. Samples were washed again in distilled water and mounted in buffered glycerin jelly.

Samples were kept at −20 °C until light microscopic examination, then observation was carried out, and slides were documented with the aid of a Nikon Optiphot 2 Photomicroscope equipped with an epifluorescent illumination source. Filipin stains free cholesterol companied by shiny silver fluorescence. Nile blue stains phospholipids and fatty acids with a dark blue color and also produces strong red fluorescence in the presence of neutral lipids. Diazo dye Oil red stains neutral fat by deep red color.

### 4.6. Electron Microscopy

For electron microscopy, 2 µm thick tissue pieces were cut with sharp blades from randomly selected lobes of livers removed from the experimental animals and transferred immediately to the mixture of 4% paraformaldehyde and 2.5% glutaraldehyde. Then, a post-fixation step, a 2 h incubation of samples in ice-cold 2% osmium tetroxide solution, diluted in 0.1 M phosphate buffer, was carried out. Blocks were dehydrated in rising ethanol series, then transferred to propylene oxide, and later placed and left for 2 h in a mixture of propylene oxide and Durcupan ACM Araldite resin (Sigma Aldrich). The samples were positioned in a clear fresh resin and polymerized at 56 °C for at least 48 h. Serial ultrathin sections were cut and collected on copper grids, contrasted in drops of saturated uranyl-acetate dissolved in ethyl alcohol and further in Reynolds’ lead citrate stain. The observation was performed with a JEOL 1200EX II transmission electron microscope.

### 4.7. Cholesterol Extraction, Sample Preparation and GC-MS Measurements

Mouse feces samples were collected and kept at room temperature until mass constancy. The lipid fraction was extracted with the chloroform–methanol method [42,43,44]. A 2:1 (*v/v*) mixture of the non-polar solutions was used, and after the extraction, the solutions were dried via rotary evaporation with an Eppendorf Concentrator Plus. The extracts were re-dissolved in dichloromethane, vortexed for 30 sec and centrifuged for 10 min at 11,000 rpm. The filtered supernatant was diluted further with dichloromethane (in total tenfold dilution), and each sample contained internal cholesterol standard (with 10 μg/mL final concentration), and a derivatizing agent BSTFA + TMCS (with 50 μL/mL final concentration).

For GC-MS measurements, an Agilent Series 6890N GC-5975 MSD instrument was used with a Supelco^TM^-5MS (30 mm × 0.25 mm × 0.25 μm) column. A 2 μL sample was introduced with pulsed splitless injection, and the carrier gas was helium (1.4 mL/min). The temperature program was the following: 150 °C (0.5 min); 20 °C/min to 300 °C (0.5 min); 25 °C/min to 310 °C (5 min). For mass spectrometry measurements, scan (*m/z* 35–550) and SIM mode were used with 8 min of solvent delay [45]. Cholesterol (458 M+) was detected at 10.768 min, and 5-alpha-cholestane (372 M+) at 9.406 min retention time.

### 4.8. Statistical Analysis

Data were analyzed using two-sided unpaired *t*-tests. Values are expressed as mean ± SEM. A value of *p* < 0.05 was considered statistically significant. * *p* < 0.05; ** *p* < 0.01; *** *p* < 0.001. For lipidomic data, the false discovery rate (q value) was determined according to the Storey–Tibshirani method [46]; significance was accepted for *p* < 0.05, corresponding to a false discovery rate of q < 0.05. OPLS-DA on lipidomic datasets was performed by MetaboAnalyst [47]. To assess the correlation between liver and plasma lipidomes, Pearson correlation analysis was applied.

## Data Availability

Not applicable.

## Supplementary Materials

The following supporting information can be downloaded at: https://www.mdpi.com/article/10.3390/ijms231911274/s1.
