# Supplementary figures and images for: Remodeling of Liver and Plasma Lipidomes in Mice Lacking Cyclophilin D
Source: Int J Mol Sci. 2022 Sep 24;23(19):11274. doi: 10.3390/ijms231911274 (PMC9569465; doi:10.3390/ijms231911274)

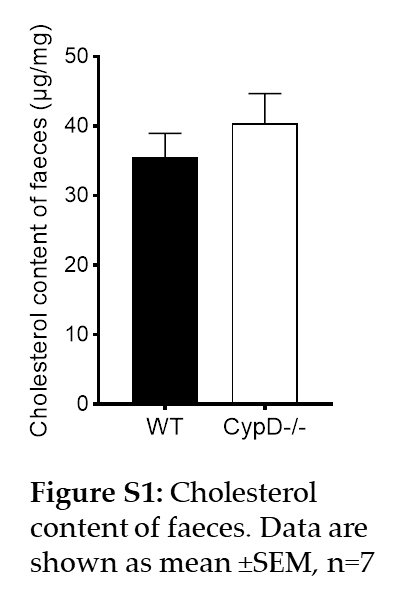

Supplement: Supplementary file 1 [file ijms-23-11274-s001.zip › Figure S1_leg.tif]
